# Long-term outcomes after surgical resection in patients with stage IV colorectal cancer: a retrospective study of 129 patients at a single institution

**DOI:** 10.1186/s12957-019-1599-3

**Published:** 2019-03-23

**Authors:** Makoto Sudo, Shinji Furuya, Hiroki Shimizu, Yuuki Nakata, Hiroshi Iino, Kensuke Shiraishi, Hidenori Akaike, Naohiro Hosomura, Yoshihiko Kawaguchi, Hidetake Amemiya, Hiromichi Kawaida, Shingo Inoue, Hiroshi Kono, Daisuke Ichikawa

**Affiliations:** First Department of Surgery, Faculty of Medicine, University of Yamanashi, 1110 Shimokato, Chuo, Yamanashi 4093898 Japan

**Keywords:** Colorectal cancer, Stage IV, Surgical resection, Outcome, Metastasis

## Abstract

**Background and purpose:**

Approximately 20% of all patients with colorectal cancer (CRC) are diagnosed at more advanced stages with synchronous distant metastasis, and the prognosis in these patients is usually poor. The aim of this study was to determine the factors that can identify subgroup(s) of patients with stage IV CRC who could benefit from curative (R0) resection of both primary and metastatic lesions.

**Patients and methods:**

A total of 126 patients with stage IV CRC who underwent surgical resection of primary tumor were retrospectively analyzed. Among these patients, 26 cases of R0 resection were further examined subsequently. Information on various clinicopathological factors of the patients were obtained from hospital records. Overall survival was estimated using the Kaplan-Meier method, and log-rank tests were used to compare survival distribution. All the factors with *P* < 0.05 in univariate analysis were analyzed in the Cox proportional hazards model.

**Results:**

CEA negativity, left-sided tumor, R0 resection, differentiated histology, and nodal staging less than N1 were independent factors that predicted better prognosis in all the 126 patients with stage IV CRC. Tumor depth of T3 or less was significantly correlated with better survival in patients who had undergone R0 resection.

**Conclusion:**

Our findings demonstrate that it is possible to select patients in whom surgical resection would yield better prognosis, from a variety of patient subgroups with stage IV CRC.

## Background

Recent advances in diagnostic tools and the widespread availability of routine medical screening have provided considerable opportunities for detecting colorectal cancers at a relatively early stage. In contrast, approximately 20% of all patients with colorectal cancer are diagnosed at more advanced stages with synchronous distant metastasis. The prognosis in these patients is usually poor [1, 2].

However, there is heterogeneity in patients with stage IV CRC. Some patients may have a single liver metastasis, and the other may present with multiple metastatic lesions. Surgical interventions are indispensable in some patients with severe tumor-related symptoms such as bowel obstruction, perforation, and bleeding. Resection of both primary and metastatic lesions may be a choice of treatment when it can be done in curative intent. Moreover, recent advances in chemotherapeutic agents have ushered in new strategies, and chemotherapeutic therapies without primary tumor resection provide better prognosis in stage IV CRC patients with unresectable metastatic lesions [3]. So, appropriate curative or palliative therapeutic plan must be made for each stage IV CRC patients concerning sites of disease, extent of metastasis, symptoms, performance status, and comorbidities of the patients.

At our institute, aggressive surgical resection of both primary and metastatic lesions has been the standard of care in patients with stage IV CRC. However, we have encountered patients who develop early recurrence after macroscopic curative resection and have extremely poor prognosis. This finding implies that while some subgroup(s) of patients may considerably benefit from surgical resection in curative intent, other subgroups may more likely benefit from chemotherapy-oriented strategies. Given the above, it is essential to tailor therapeutic strategies for each patient to obtain optimum results.

Therefore, in this study, we firstly analyzed clinicopathological factors in patients with stage IV CRC who underwent surgical resection of the primary tumor at our institute during the last decade and assessed the correlation between clinicopathological factors and prognosis. Secondly, we determined the factors that can identify subgroup(s) of patients with stage IV CRC who can benefit from surgical resection of both primary and metastatic lesions.

## Patients and methods

A total of 129 patients with stage IV colorectal tumors underwent surgical resection of the primary tumor at the University of Yamanashi hospital from 2001 to 2012. Patients with tumors arising from the appendix or the anal canal were excluded as were those with other specific histological types, such as neuroendocrine tumors and gastrointestinal stromal tumors. Thus, the data from 126 patients who underwent surgical resection of primary colorectal cancer were used in this retrospective study. None of the patients had received preoperative chemotherapy and/or radiation therapy, and surgical resection and lymphadenectomy were performed according to the colorectal cancer treatment guidelines issued by the Japanese Society for Cancer of the Colon and Rectum [4].

Information on various clinicopathological factors of the patients, including age, gender, surgical procedures, histological type, depth of tumor invasion, status of pathological lymph node metastasis, status of distant metastatic organs, and tumor markers were obtained from hospital records and were retrospectively analyzed. The location of the primary lesion(s) was also analyzed as a predictor of postoperative prognosis. Histological type was categorized as either differentiated (diff; well or moderately differentiated tubular adenocarcinoma) or undifferentiated (undiff; mucinous or poorly differentiated adenocarcinoma). Regarding the tumor location, those arising from the cecum, and the ascending and the transverse colon (up to the splenic flexure), were defined as right-sided tumors, whereas those arising from the descending colon to the rectum were defined as left-sided tumors. Colorectal tumors were macroscopically and microscopically classified based on the Union for International Cancer Control (UICC) criteria, seventh edition [5].

Chemotherapy regimens were selected according to Japanese guidelines [5], and the follow-up program consisted of physical examination, hematological and blood chemistry panels, and blood tests for CEA and CA19-9 at every 3 months. Abdominal computed tomography (CT) was performed every 4 to 6 months, and the patients were prescribed adjuvant chemotherapy, such as FOLFOX, FOLFIRI, or CAPEOX, even after a curative (R0) resection. The assessments described above were repeated every month.

### Statistical analysis

Overall survival (OS) was estimated using the Kaplan-Meier method, and log-rank tests were used to compare survival distribution. A *P* value of < 0.05 was considered statistically significant. All the factors with *P* < 0.05 in univariate analysis were analyzed in the Cox proportional hazards model. EZR version 3.2.2 was used for the statistical analysis [6].

## Results

The clinicopathological features of the patients are shown in Table 1. Of note, there were two T1 cases, while most of the patients had T3 or T4 cancers. No patients were categorized as N3, and there were 71 cases of M1a. Twenty-six patients underwent R0 resection, and half of then underwent resection of the liver tumor(s). Other metastatic sites that were resected include such as the paraaortic lymph nodes, the ovary, and the lung.

**Table 1:**
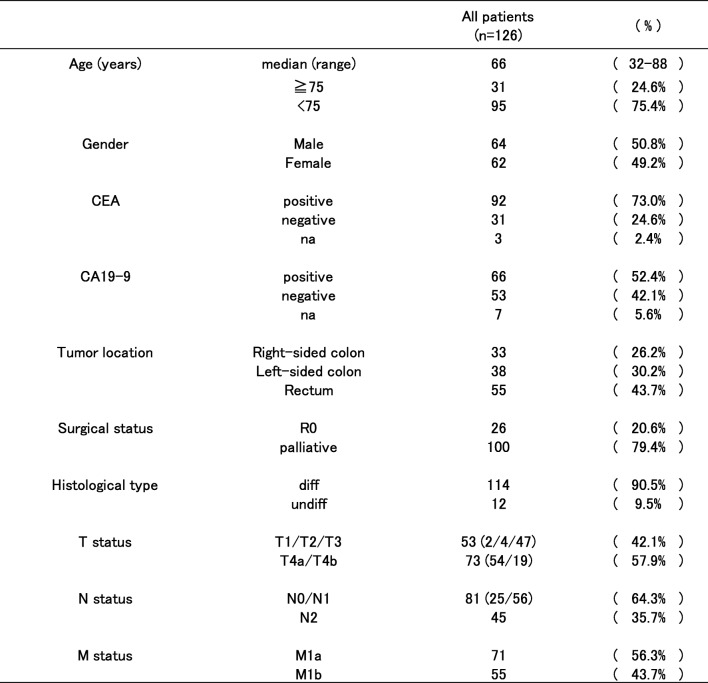
Clinicopathological features of the patients (*n* = 126)

The changes made were colored in red

*na* not available

Table 2 shows the details of the metastatic sites in all 126 patients. The most common metastatic site was the liver (94 cases, 74.6%), followed by the lung (42 cases, 33.3%), and most of the cases with lung metastasis were M1b.

**Table 2:**
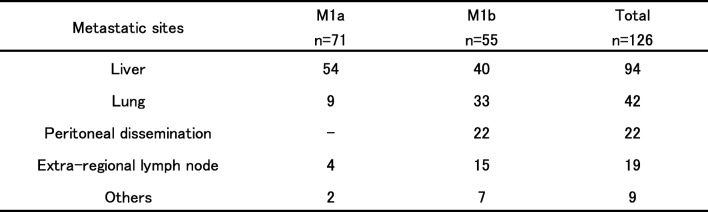
Metastatic sites

**Fig. 1:**
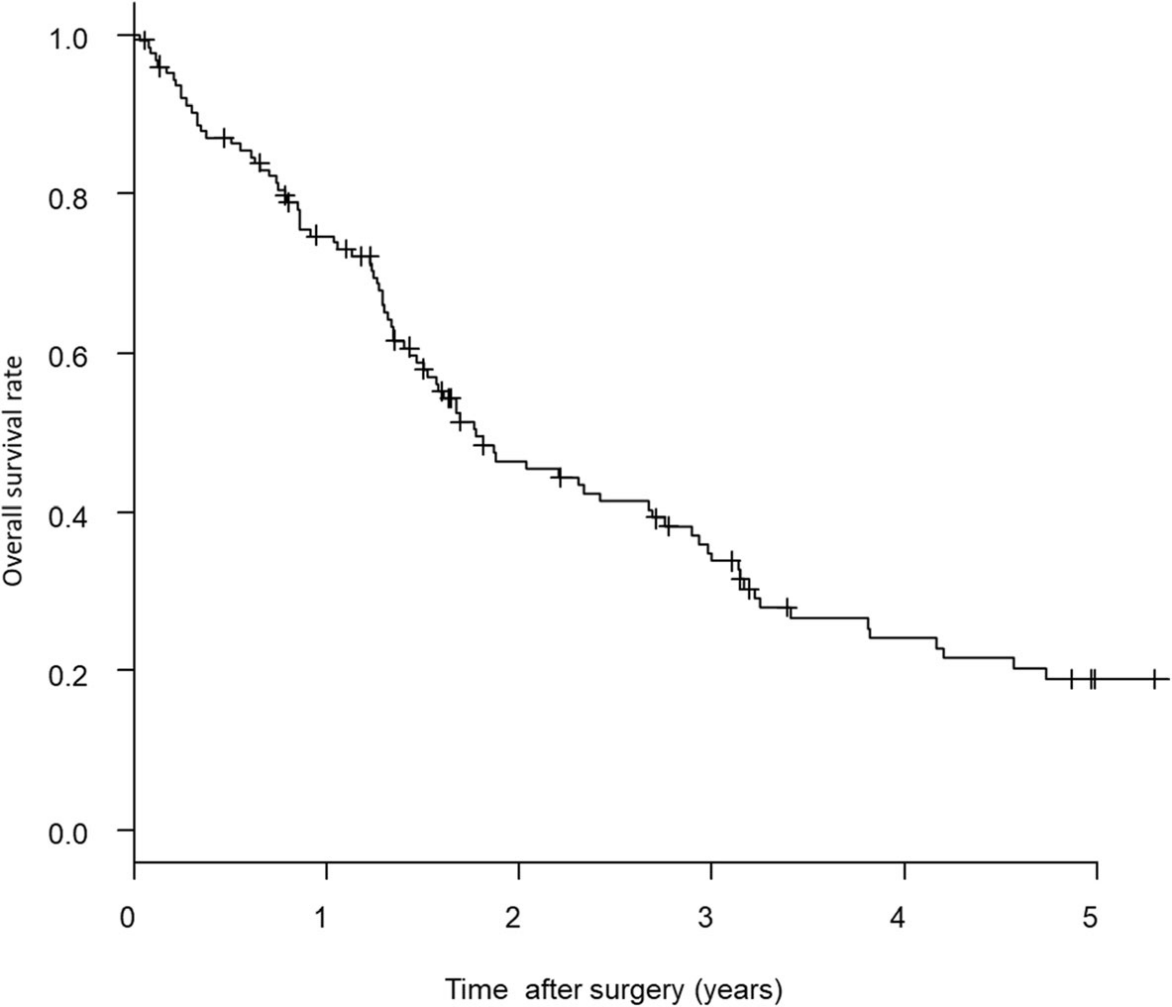
Five-year overall survival of all the 126 cases

With respect to prognosis, the median follow-up period was 18.4 months (range, 0.37–131.5 months), and the 5-year overall survival (OS) in all the 126 cases was 19.1% (Fig. 1). Multiple clinicopathological factors, except age and sex, were found to be statistically significant prognostic factors associated with OS in univariate analysis (Table 3).

**Table 3:**
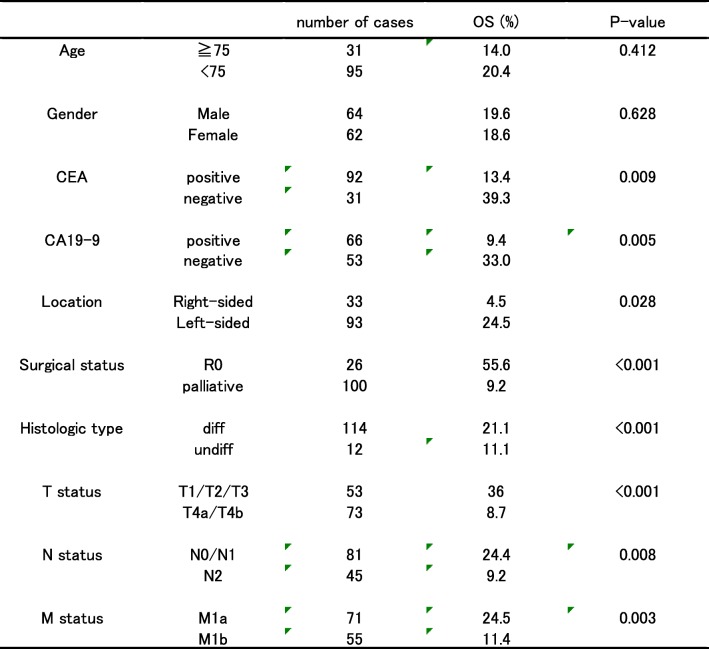
Univariate analysis of prognostic variables that correlated with OS (5 years)

However, multivariate analysis revealed that CEA negativity, left-sided tumor, R0 resection, differentiated histology, and nodal staging less than N1 were independent factors that predicted better prognosis. T and M status, along with preoperative serum CA19-9 level, were not significantly correlated with prognosis (Table 4).

**Table 4:**
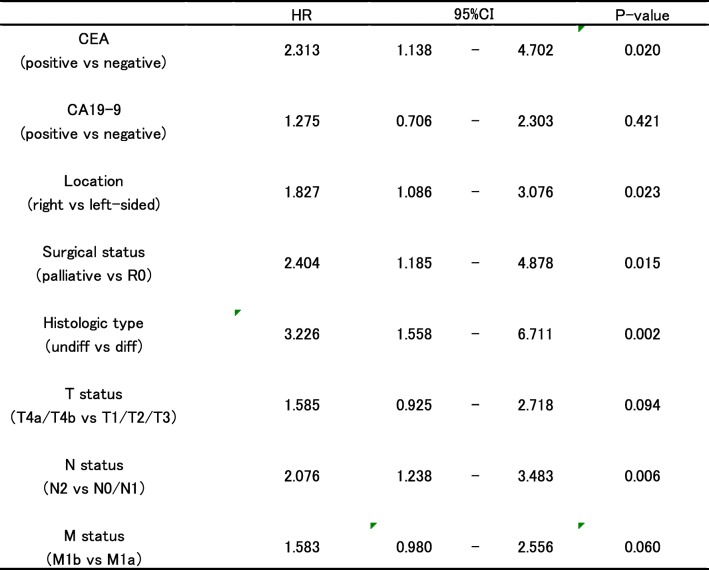
Multivariate analysis of prognostic variables that correlated with OS (5 years)

*HR* hazard ratio, *CI* confidence interval

As the purpose of this study is to determine the factors that can identify subgroup(s) of patients with stage IV CRC who can benefit more from curative resection of both primary and metastatic lesions, further prognostic analyses were performed only in a subgroup of patients who had undergone R0 resection.

**Fig. 2:**
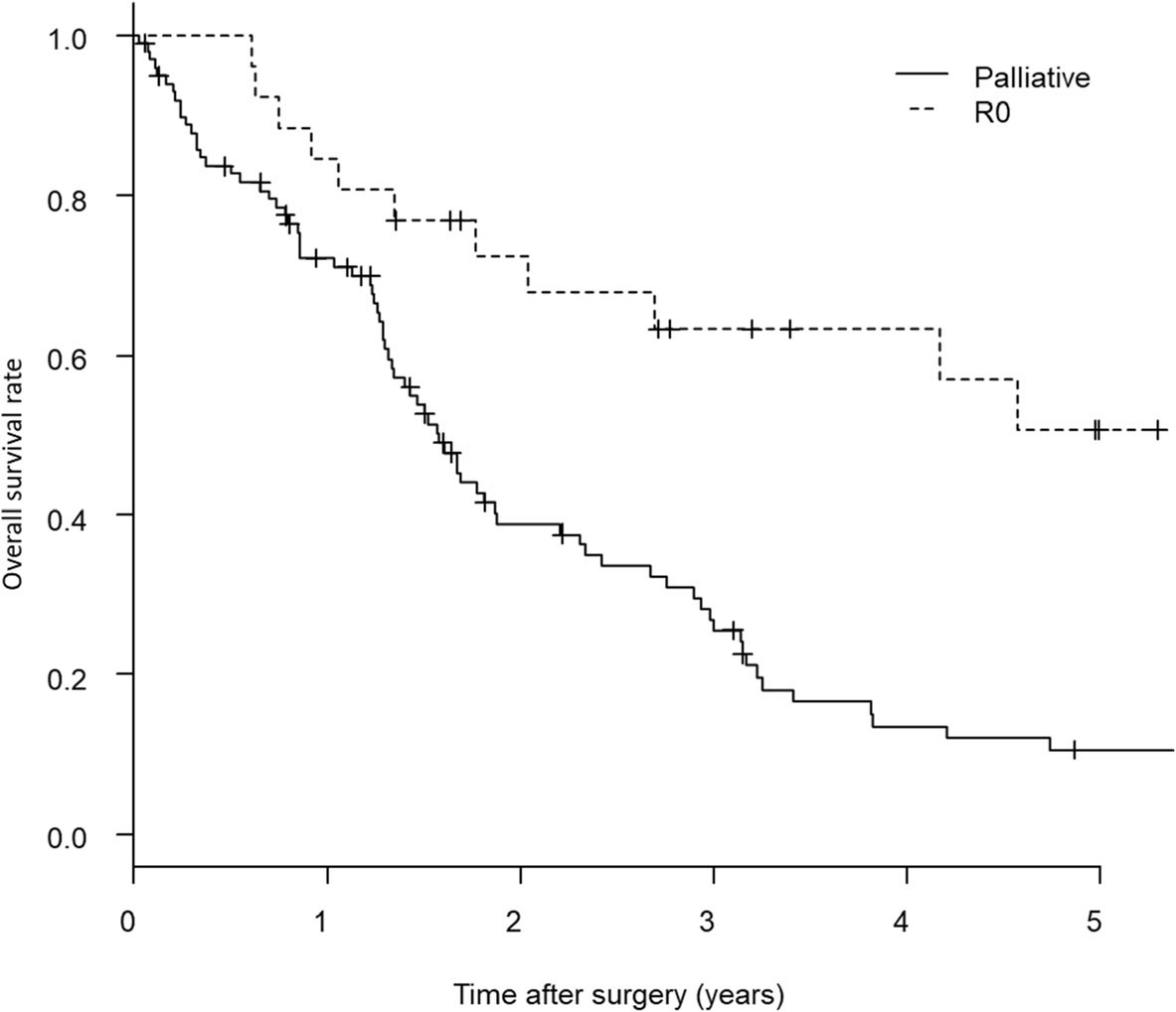
Comparison of 5-year overall survival between patients with R0 resection and palliative resection. Patients with R0 resection (*n* = 26) showed statistically significant better survival than that of the palliative resection (*n* = 100) by Kaplan-Meier survival method and log-rank test (5-year OS: R0 resection vs. palliative resection = 55.6% vs. 9.2%, *P* < 0.001)

R0 resection was identified as one of the most significant prognostic factors in multivariate analysis, and patients in whom R0 resection could be achieved showed good prognosis (Fig. 2).

Although age, depth of tumor invasion, and tumor location were also identified as significant prognostic factors during univariate analysis of 26 cases of R0 resection patients, only tumor depth of T3 or less was significantly correlated with better survival in multivariate analysis (Table 5).

**Table 5:**
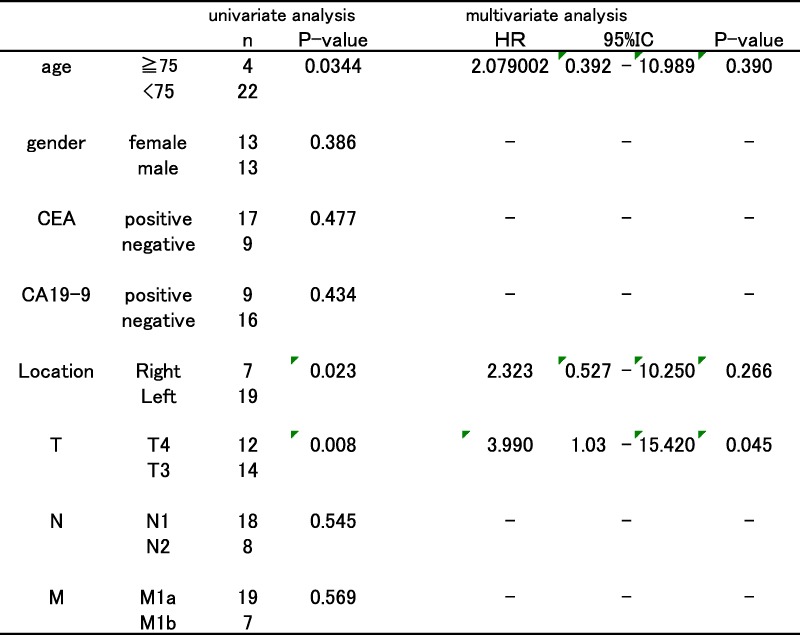
Univariate and multivariate analysis of 26 cases of R0 resection

*HR* hazard ratio, *CI* confidence interval

## Discussion

In a retrospective analysis of stage IV CRC, we firstly show that CEA negativity, R0 resection, differentiated histology, nodal staging less than N1, and left-sided tumor are independent factors that predicted better prognosis.

Recently, other groups have analyzed prognostic factors in patients with stage IV CRC and have demonstrated that high pT stage and presence of positive node(s) are significant negative prognostic factors [7, 8]. Moreover, the effect of the tumor location on drug sensitivity and prognosis in unresectable disease has recently attracted attention. Specifically, the incidence of right-sided colon cancer has been reported to have increased in Western countries [9, 10], and several recent retrospective studies have reported that patients with right-sided tumors have a worse prognosis than those with left-sided CRC did [11, 12]. The underlying causes for this observed variation in clinical features are thought to be the differences in their molecular profiles [13]. Our result is in agreement with these previous studies.

Secondly, our study also revealed that CEA negativity, nodal staging, and sideness of tumor are not the independent prognostic factors in patients with stage IV colorectal cancer who had undergone curative resection. Similarly, Ishihara et al. (2014) have reported that the prognostic value of tumor location may differ among patient subgroups; specifically, they have demonstrated that right-sided tumors were associated with a significantly worse prognosis after palliative resection but not after R0 resection [12]. These findings indicate that radical resection may play a more crucial role in determining prognosis than biological differences in tumor location. In this study, only the depth of tumor invasion showed correlation with prognosis after R0 resection. This finding may imply that tumor invasion can better reflect biological aggressiveness of tumors than other factors, such as N status or histology, and that tumor with T3 or less invasion is a good candidate for aggressive curative resection of both primary and metastatic tumors.

Finally, the validity of primary tumor resection in asymptomatic patients with incurable stage IV colorectal cancer remains unclear [14]. Several studies have demonstrated that, compared to chemotherapeutic strategies, resection of primary lesions is not associated with improvement in overall survival in patients with stage IV colorectal cancer [15, 16]. Moreover, there is a hypothesis pointing out that removal of the primary tumor can promote growth of metastases by removing putative metastatic-inhibiting factors or immune responses promoted by the primary tumor [17]. Clinicopathological factors relating to poor prognosis, such as CEA positivity, undifferentiated histology, nodal staging more than N2, and left-sided tumor, might fit with this hypothesis.

This study has certain limitations, such as low patient number and the fact that it is a retrospective single-institute study. Thus, further large and multi-institutional studies are needed to confirm these findings.

## Conclusions

Our findings demonstrate that it is possible to select patients in whom aggressive surgical resection would yield better prognosis, from a variety of patient subgroups with stage IV CRC. Furthermore, our results also support aggressive surgical resection of primary tumors that have invaded the colorectal wall (T3 or less) in patients with stage IV CRC.
